# Angiopoietin-1 induces survival and proliferation of hair follicle dermal papilla cells through integrin α5β1 signaling

**DOI:** 10.3389/fmed.2025.1649763

**Published:** 2025-09-24

**Authors:** Jang-Hyuk Yun

**Affiliations:** College of Veterinary Medicine and Institute of Veterinary Science, Kangwon National University, Chuncheon, Republic of Korea

**Keywords:** angiopoietin-1, human follicle dermal papilla cells, integrin α5β1, survival, proliferation, alopecia

## Abstract

**Background:**

Androgenetic alopecia is a common form of hair loss primarily mediated by dihydrotestosterone (DHT), which induces apoptosis and inhibits proliferation in dermal papilla cells (DPCs). Current treatments, such as minoxidil and finasteride, often show limited efficacy and can cause adverse effects, underscoring the need for safer and more targeted therapies.

**Methods:**

This study investigated the protective and proliferative effects of angiopoietin-1 (Ang1) on human follicle dermal papilla cells (HFDPCs) under DHT-induced stress. Apoptosis and proliferation were assessed using flow cytometry and BrdU assays. Western blotting was used to examine intracellular signaling pathways. The expression and functional relevance of Tie and integrin receptors were evaluated using gene expression analysis and blocking antibodies.

**Results:**

Ang1 significantly reduced DHT-induced apoptosis and restored proliferation in HFDPCs. These effects were mediated via activation of the PI3K/AKT and MAPK/ERK1/2 pathways through integrin α5β1. Neither Tie-1 nor Tie-2 receptors were detected in HFDPCs, indicating that Ang1 acts through a Tie-2-independent mechanism. Given the well-established role of Ang1 in promoting vascular stability via the Tie-1–Tie-2 axis in endothelial cells, it is plausible that Ang1 may also support follicular health indirectly by enhancing perifollicular vascularization.

**Conclusion:**

Ang1 enhances HFDPC survival and proliferation through integrin α5β1-mediated signaling. In addition to its direct protective effects on DPCs, Ang1 may promote angiogenic support in the hair follicle microenvironment. These findings position Ang1 as a potential dual-action therapeutic candidate for androgenetic alopecia.

## 1 Introduction

Androgenetic alopecia, often referred to as male pattern baldness, is characterized by the miniaturization of hair follicles owing to the influence of dihydrotestosterone (DHT), a potent derivative of testosterone that transforms thick terminal hairs into thin vellus hairs (1). Without treatment, the condition results in gradual and progressive hair thinning (2). This is the leading cause of hair loss, and its incidence tends to increases with age (3–5). Male pattern baldness can negatively impact mental well-being, contributing to issues like persistent self-consciousness, age-related anxiety, and a lack of energy, affecting individuals of both sexes (6–8). Currently, the U. S. Food and Drug Administration (FDA) has approved minoxidil (MNX) and finasteride as treatments for hair loss. Nevertheless, even with the use of these treatments, many individuals continue to struggle with hair loss. Moreover, MNX may cause adverse reactions such as itching and contact dermatitis (9), while the side effects of finasteride include headaches, dizziness, skin irritation, and sexual dysfunction (10–13). Accordingly, there is a growing need for alternative hair loss therapies that offer both effectiveness and a reduced risk of side effects.

The hair follicle, an epidermal appendage, consists of both epidermal and dermal sections. At the base of the follicle lies the dermal papilla, a crucial dermal component essential for hair follicle development and growth cycles (14–17). Notably, the DP has the unique capacity to initiate new hair follicle formation and regulate the number of matrix cells, influencing hair size and density (18–23). Thus, it is proposed that enhancing the number of DP cells or inhibiting their apoptosis may contribute to preventing hair loss by promoting hair thickness and density. Vascular endothelial growth factor (VEGF), a key angiogenic factor primarily recognized for stimulating blood vessel formation, has also been shown to directly increase DP cell proliferation in hair follicles (24). However, the effects of other angiogenic factors on DP cells remain largely unexplored, warranting further investigation.

Angiopoietin-1 (Ang1) is a protein that plays an important role in vascular development and angiogenesis along with VEGF. Both Ang1 and VEGF are angiogenic factors that are involved in survival, proliferation, migration, and tube formation by activating AKT or ERK1/2 in various types of endothelial cells (25–29). Ang1 and VEGF bind to Tie-2 receptor and integrin or VEGF receptors, respectively, transmit downstream signaling, and perform various actions including angiogenesis (26–28, 30–33). Although Ang1 and VEGF act specifically on endothelial cells, VEGF is known to be involved in the survival or proliferation of various cells including DP cells (24, 34–36). However, the effect of Ang1 on DP cells is completely unknown. Therefore, we aimed to investigate the effect of Ang1 on DP cells and the related mechanism.

In this study, it was demonstrated that Ang1 prevented the survival and proliferation of human follicle dermal papilla cells (HFDPCs) decreased by DHT. In addition, Ang1 induced the survival of HFDPCs through the AKT pathway and the proliferation through the ERK1/2 pathway. In addition, Ang1 was found to be involved in the survival and proliferation of HFDPCs through integrin α5β1, not Tie-2. These results suggest that the Ang1/integrin α5β1 axis can be a potential treatment for androgenetic alopecia by preventing the survival and proliferation of HFDPCs decreased by DHT.

## 2 Materials and methods

### 2.1 Cell cultures

Human follicle dermal papilla cells (PromoCell, Heidelberg, Germany) and human dermal microvascular endothelial cells (PromoCell) were cultured in a follicle dermal papilla cell growth medium and endothelial cell growth medium (both from PromoCell), respectively. The cells were incubated at 37 °C in a humidified atmosphere containing 5% CO_2_.

### 2.2 Reagents and antibodies

Recombinant human Ang1, Ang2, and VEGF were purchased from R&D Systems (Minneapolis, MA, USA). MTT (3-(4,5-dimethylthiazol-2-yl)-2,5-diphenyltetrazolium bromide), Wortmannin, PD98059, SB202190, anti-Tie-1, anti-Tie-2, Gly-Arg-Gly-Asp-Ser (GRGDS) peptide, and functional blocking antibodies against integrins α1–6, αv, β1, and α5β1 were purchased from Millipore-Sigma (St. Louis, MO, USA). Other reagents and antibodies used were: anti-cleaved caspase-3, anti-Bax, anti-Bcl-2, anti-Bcl-xL, anti-phospho-AKT, anti-AKT, anti-phospho-ERK1/2, anti-ERK1/2, anti-phospho-p38, anti-p38, anti-integrin α4, anti-integrin α5, anti-integrin αv, anti-integrin β1, anti-integrin β3, anti-integrin β4, and anti-integrin β5 (Cell signaling Technology, Danvers, MA, USA), anti-β-tubulin and peroxidase-conjugated secondary antibodies (Santa Cruz Biotechnology, Dallas, TX, USA), Muse® Annexin V & Dead Cell Assay Kit (FITC) (Luminex Corporation, Austin, TX, USA), and 5′-bromodeoxy-2′-uridine (BrdU) cell proliferation ELISAs (Roche, Indianapolis, IN, USA).

### 2.3 Cell viability assay

Cell viability was measured using the MTT assay kit (Millipore-Sigma). About 5 × 10^3^ cells were plated in 96-well plates for 24 h and treated with indicated reagents for 48 h. Thereafter, the cells were treated with 100 μL of MTT (5 mg/mL) for 3 h. The formazan levels were measured using the absorbance at 570 nm.

### 2.4 Apoptosis assay

The apoptotic effect was assessed using the Annexin-V-FITC/PI double-staining assay, following the instructions provided by the manufacturer (Muse® Annexin V & Dead Cell Assay Kit). Cells (3 × 10^5^) were treated with the indicated agents for 48 h. Post incubation, cells were collected in 1 mL of medium containing 1% fetal bovine serum (FBS). Subsequently, the cell suspension (100 μL) was mixed with Muse® Annexin V & Dead Cell reagent and vortexed for 5 s. The mixture was then incubated for 20 min at room temperature and analyzed using the Muse™ Cell Analyzer. Data were processed using the Muse Analysis Software, and cells positive for annexin-V only or annexin-V/PI double staining were considered apoptotic. Each experiment was conducted in triplicate.

### 2.5 Western blot analysis

Cells were harvested and lysed in radioimmunoprecipitation assay (RIPA) buffer (Thermo Fisher Scientific, Waltham, MA, USA) supplemented with a protease and phosphatase inhibitor cocktail (Thermo Fisher Scientific). Protein concentration was determined using the bicinchoninic acid (BCA) assay (Thermo Fisher Scientific), and equal amounts of protein (30 μg per lane) were resolved on 10–12% SDS–polyacrylamide gels. Following electrophoresis, proteins were transferred onto nitrocellulose membranes (0.45 μm pore size; GE Healthcare, Chicago, USA) using a semi-dry blotting system (Bio-Rad, Hercules, CA, USA). Membranes were blocked in 5% non-fat dry milk diluted in TBST buffer (Tris-buffered saline with 0.1% Tween-20) for 1 h at room temperature. Primary antibodies were diluted (1: 1000 in 5% BSA in TBST) and incubated overnight at 4 °C. Following three washes with TBST (10 min each), membranes were incubated with HRP-conjugated anti-rabbit or anti-mouse secondary antibodies (Santa Cruz Biotechnology, diluted 1: 5,000 in 5% non-fat dry milk/TBST) for 1 h at room temperature. Bands were visualized using an enhanced chemiluminescence (ECL) detection reagent (Thermo Fisher Scientific) and imaged using the ImageQuant LAS 500 system (GE Healthcare).

### 2.6 BrdU ELISA proliferation assay

To measure cell proliferation, a Cell Proliferation BrdU ELISA kit (Roche) was used according to the manufacturer’s protocol. Cells treated with the indicated agents for 48 h were labeled with 10 μM BrdU for 1 h. The anti-BrdU peroxidase conjugated antibody was then incubated for 90 min. After washing, the bound peroxidase was detected based on the substrate reaction, which was measured at 450 nm.

### 2.7 Real-time quantitative PCR

All RNA was extracted from cells and tissues using the RNeasy Plus Mini kit (Qiagen). cDNAs were generated from RNAs (1 μg) using 2.5 μM oligo-dT primers, 1 mM dNTPs, and MuLV reverse transcriptase. qRT-PCR assays were performed in the qPCR Master Mix for SYBR Green PCR Master Mix (Applied Biosystems). qRT-PCR was performed using the following primers: *TIE2* (forward: 5’-GCTTGCTCCTTTCTGGAACTGT-3′ and reverse: 5′- CGCCACCCAGAGGCAAT-3′); *TIE1* (forward: 5’-AGAACCTAGCCTCCAAGATT-3′ and reverse: 5’-ACTGTAGTTCAGGGACTCAA-3′); *ITGA4* (forward: 5’-GCTTCTCAGATCTGCTCGTG-3′ and reverse: 5’-GTCACTTCCAACGAGGTTTG-3′); *ITGA5* (forward: 5’-TGCAGTGTGAGGCTGTGTACA-3′ and reverse: 5’-GTGGCCACCTGACGCTCT-3′); *ITGAV* (forward: 5’-AATCTTCCAATTGAGGATATCAC-3′ and reverse: 5’-AAAACAGCCAGTAGCAACAAT-3′); *ITGB1* (forward: 5’-GAAGGGTTGCCCTCCAGA-3′ and reverse: 5’-GCTTGAGCTTCTCTGCTGTT-3′); *ITGB3* (forward: 5’-CCGTGACGAGATTGAGTCA-3′ and reverse: 5’-AGGATGGACTTTCCACTAGAA-3′); *ITGB4* (forward: 5’-AGACGAGATGTTCAGGGACC-3′ and reverse: 5’-GGTCTCCTCTGTGATTTGGAA-3′); *ITGB5* (forward: 5’-GGAGCCAGAGTGTGGAAACA-3′ and reverse: 5’-GAAACTTTGCAAACTCCCTC-3′); and *ACTB* (forward: 5’-GGGAAATCGTGCGTGACATT-3′ and reverse: 5’-AGTTTCGTGGATGCCACAGG-3′). A mean quantity was estimated from triplicate qRT-PCR reactions following normalization to the control gene.

### 2.8 Statistical analysis

Statistical analyses were performed using the GraphPad Prism software (GraphPad. Inc., La Jolla, CA, USA). Depending on the experimental design, unpaired two-tailed Student’s *t*-test (assuming unequal variances), one-way analysis of variance, or two-way analysis of variance followed by Tukey’s post-hoc test was used. A *p*-value of less than 0.05 was considered statistically significant. All quantitative data are presented as the mean ± standard deviation.

## 3 Results

### 3.1 Ang1 increases survival and proliferation in HFDPCs

**Figure 1:**
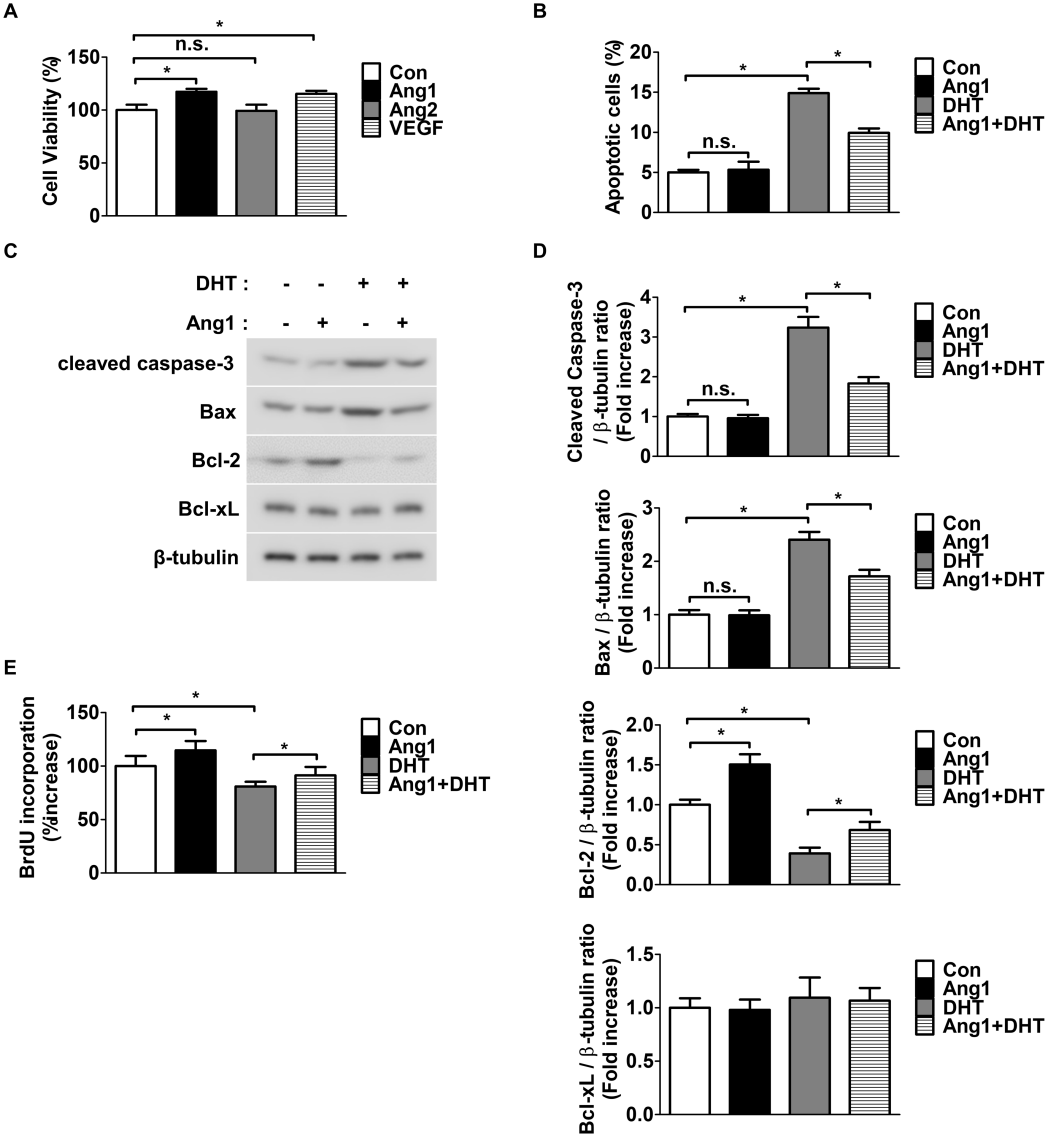
Ang1 induces survival and proliferation in HFDPCs. **(A)** HFDPCs were treated with Ang1 (300 ng/mL), Ang2 (300 ng/mL), and VEGF (10 ng/mL) for 48 h. The cell viability was analyzed via MTT assay. Bar graph represents mean ± SD (*n* = 5). *p < 0.05 by one-way ANOVA. **(B–E)** HFDPCs were treated with Ang1 (300 ng/mL) and/or DHT (2 μM) for 48 h. **(B)** Apoptosis of HFDPCs was analyzed using annexin-V/PI staining and flow cytometry. Bar graph represents mean ± SD (*n* = 3). *p < 0.05 by two-way ANOVA. **(C)** Western blot analysis was performed on lysates from HFDPCs to detect cleaved caspase-3, Bax, Bcl-2, and Bcl-xL. β-tubulin was used as a loading control. **(D)** Quantitative densitometric analysis in **(C)** to calculate the ratio of each protein to β-tubulin (*n* = 3). n.s, not significant. *p < 0.05 by two-way ANOVA. **(E)** Cell proliferation of HFDPCs was determined by 5′-bromodeoxy-2′-uridine (BrdU) proliferation ELISA. Bar graph represents mean ± SD (*n* = 5). *p < 0.05 by two-way ANOVA.

Initially, an MTT assay was conducted to assess whether angiogenic factors, including Ang1 and Ang2, influence the viability of HFDPCs. When HFDPCs were treated with Ang1 for 48 h, similar to VEGF, it enhanced cell viability, whereas Ang2 had no effect (Figure 1A).

Next, to accurately assess whether the enhancement in cell viability induced by Ang1 was associated with cell survival or proliferation, Muse® Annexin V & Dead Cell Assay Kit-based cytometric analysis, western blot analysis, and the BrdU cell proliferation ELISA assay were conducted. Ang1 inhibited DHT-induced apoptosis in HFDPCs (Figure 1B). Similarly, Ang1 prevented DHT-induced increases in cleaved caspase-3 and proapoptotic Bax levels, as well as DHT-induced decreases in antiapoptotic Bcl-2 levels in HFDPCs (Figures 1C,D). However, neither ang1 nor DHT affected antiapoptotic Bcl-xL levels in HFDPCs (Figures 1C,D). In addition, Ang1 increased the proliferation of HFDPCs even when treated alone and prevented the decrease in HFDPCs proliferation caused by DHT (Figure 1E). These results demonstrate that Ang1 prevents DHT-induced increase in apoptosis and decrease in proliferation of HFDPCs.

### 3.2 Ang1 induces survival through the AKT pathway and proliferation through the ERK1/2 pathway in HFDPCs

Next, the mechanisms through which Ang1 promotes survival and proliferation in HFDPCs were explored. Ang1 is widely recognized for activating AKT, ERK1/2, and p38 in endothelial cells (37, 38), with these signaling pathways playing a key role in survival or proliferation (37, 39). Based on this, it was hypothesized that Ang1 may similarly regulate survival or proliferation in HFDPCs through these pathways.

**Figure 2:**
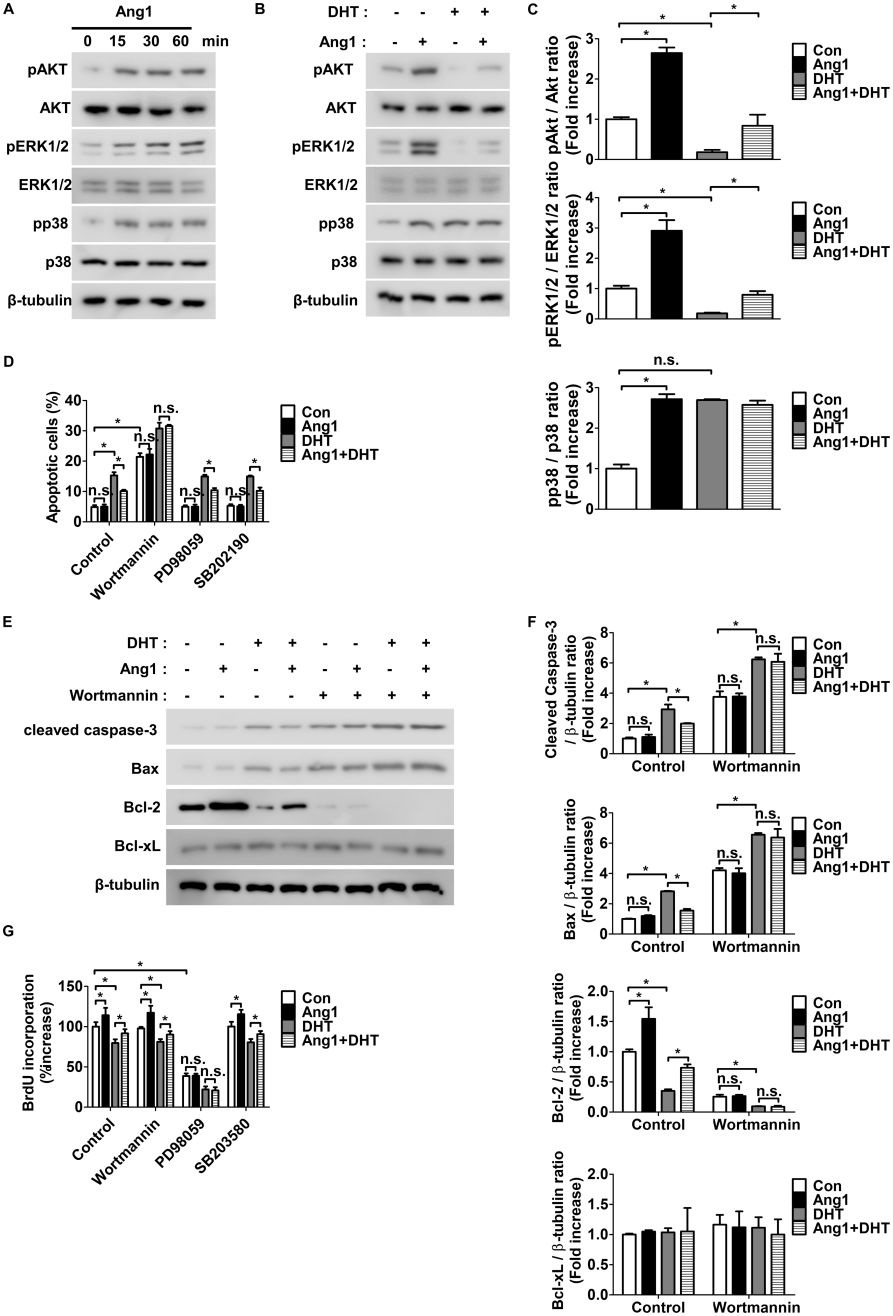
Ang1 induces survival and proliferation of HFDPCs by activating AKT and ERK1/2, respectively. **(A)** Western blot analysis for pAKT, AKT, pERK1/2, ERK1/2, pp38, and p38 were performed on lysates obtained from HFDPCs treated with Ang1 (300 ng/mL) for the indicated times. β-tubulin was used as a loading control. **(B)** Western blot analysis for pAKT, AKT, pERK1/2, ERK1/2, pp38, and p38 were performed on lysates obtained from HFDPCs treated with Ang1 (300 ng/mL) and/or DHT (2 μM) for 30 min. β-tubulin was used as a loading control. **(C)** Quantitative densitometric analysis in (B) to calculate the ratio of each protein to β-tubulin (*n* = 3). n.s, not significant. *p < 0.05 by two-way ANOVA. **(D–G)** HFDPCs preincubated with Wortmannin (1 μM), PD98059 (25 μM), or SB202190 (10 μM) for 1 h and then treated with Ang1 (300 ng/mL) and/or DHT (2 μM) for 48 h. **(D)** Apoptosis of HFDPCs was analyzed using annexin-V/PI staining and flow cytometry. Bar graph represents mean ± SD (*n* = 3). n.s, not significant. *p < 0.05 by two-way ANOVA. **(E)** Western blot analysis was performed on lysates from HFDPCs to detect cleaved caspase-3, Bax, Bcl-2, and Bcl-xL. β-tubulin was used as a loading control. **(F)** Quantitative densitometric analysis in (E) to calculate the ratio of each protein to β-tubulin (*n* = 3). n.s, not significant. *p < 0.05 by two-way ANOVA**. (G)** Cell proliferation of HFDPCs was determined by 5′-bromodeoxy-2′-uridine (BrdU) proliferation ELISA. Bar graph represents mean ± SD (*n* = 5). n.s, not significant. *p < 0.05 by two-way ANOVA.

Ang1 enhanced the phosphorylation of AKT, ERK1/2, and p38 in HFDPCs following treatment for 15, 30, and 60 min (Figure 2A). When treated with DHT, HFDPCs exhibited reduced phosphorylation of AKT and ERK1/2, while p38 phosphorylation remained unchanged (Figures 2B,C). Furthermore, Ang1 inhibited the DHT-induced reduction in AKT and ERK1/2 phosphorylation in HFDPCs (Figures 2B,C). To investigate whether Ang1-induced activation of AKT, ERK1/2, and p38 plays a role in apoptosis or proliferation, the AKT inhibitor Wortmannin, the ERK1/2 inhibitor PD98059, and the p38 inhibitor SB202190 were utilized. Wortmannin blocked Ang1-induced AKT phosphorylation in HFDPCs with no impact on the phosphorylation of ERK1/2 or p38 (Supplementary Figure 1A). PD98059 blocked Ang1-induced ERK1/2 phosphorylation with no impact on the phosphorylation of AKT or p38 (Supplementary Figure 1A), while SB202190 blocked Ang1-induced p38 phosphorylation with no impact on the phosphorylation of AKT or ERK1/2 (Supplementary Figure 1B). Interestingly, Wortmannin fully blocked Ang1-mediated survival under DHT treatment in HFDPCs, whereas PD98059 and SB202190 had no effect (Figure 2D). Furthermore, Wortmannin fully prevented the Ang1-induced reduction in cleaved caspase-3 and pro-apoptotic Bax levels, as well as the increase in anti-apoptotic Bcl-2 levels under DHT treatment in HFDPCs (Figures 2E,F). In contrast, PD98059 fully inhibited Ang1-driven proliferation in HFDPCs, while neither Wortmannin nor SB202190 contributed to proliferation. (Figure 2G). These findings indicate that Ang1 promotes survival via the AKT pathway and drives proliferation through the ERK1/2 pathway in HFDPCs.

### 3.3 HFDPCs lack Tie-2 receptors but express a variety of integrins

**Figure 3:**
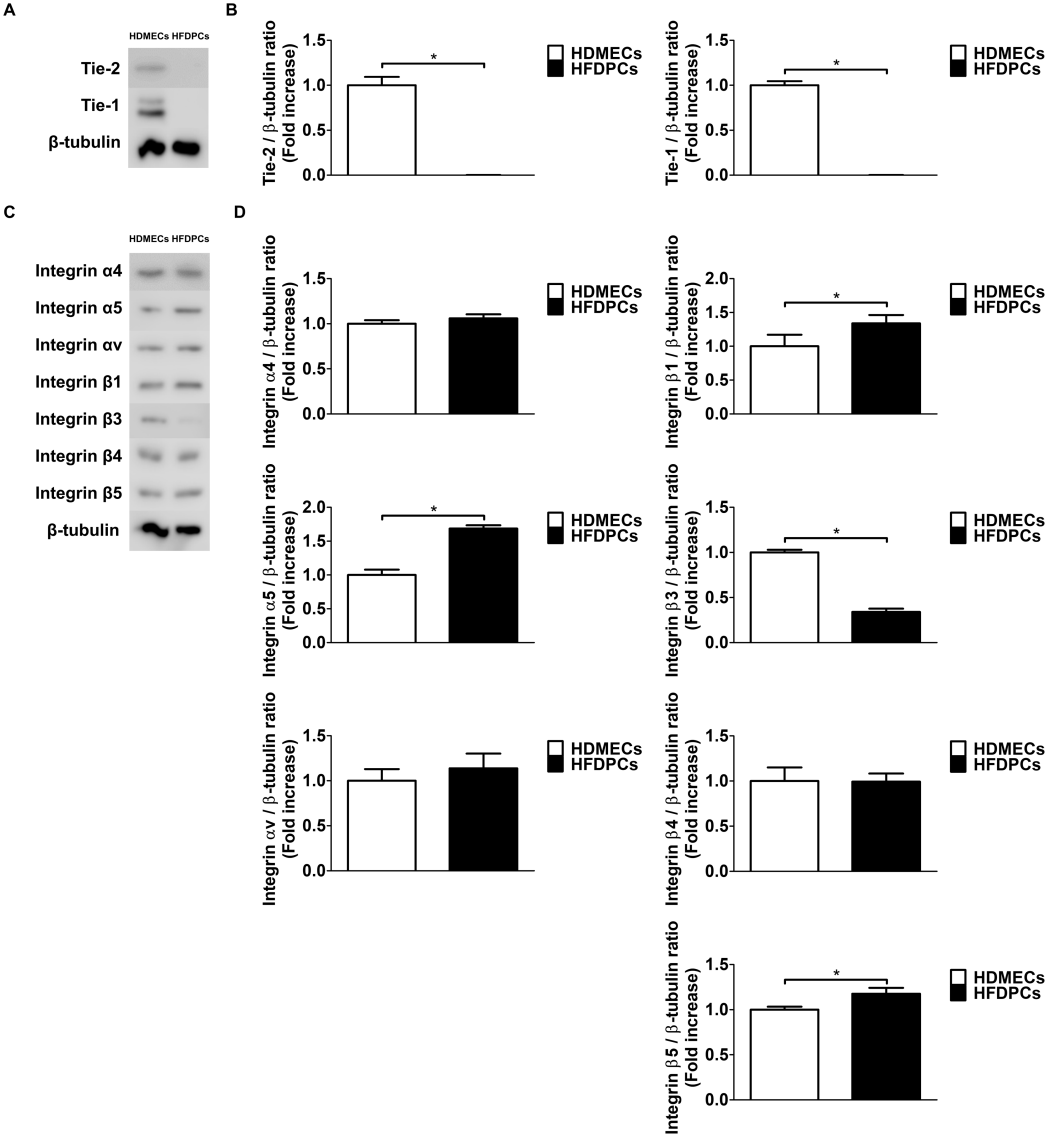
HFDPCs do not express Tie-2 receptors, but they exhibit a variety of integrin receptors. **(A)** Western blot analysis for Tie-2 and Tie-1 were performed on lysates obtained from HFDPCs and HDMECs. β-tubulin was used as a loading control. **(B)** Quantitative densitometric analysis in **(A)** to calculate the ratio of each protein to β-tubulin (*n* = 3). *p < 0.05 by Student *t* test. **(C)** Western blot analysis for Integrin α4, α5, αv, β1, β3, β4, and β5 were performed on lysates obtained from HFDPCs and HDMECs. β-tubulin was used as a loading control. **(D)** Quantitative densitometric analysis in **(C)** to calculate the ratio of each protein to β-tubulin (*n* = 3). *p < 0.05 by Student *t* test.

Western blot and qRT-PCR were subsequently performed to examine the presence of Tie-2 and integrins, which serve as receptors for Ang1, in HFDPCs. Notably, since Tie-2 is specifically and highly expressed in endothelial cells (40), HDMECs, a type of endothelial cell, were utilized. Interestingly, neither Tie-1 nor Tie-2, both members of the Tie receptor family, were expressed in HFDPCs (Figures 3A,B). Furthermore, the mRNA expression of Tie-2 and Tie-1 was nearly undetectable in HFDPCs compared to HDMECs (Supplementary Figure 2A). In contrast, integrin α4, α5, αv, β1, β3, β4, and β5 were prominently expressed in HFDPCs, with their mRNA levels also being significantly detectable (Figures 3C,D and Supplementary Figure 2B). These findings indicate that the Tie-2 receptor is absent in HFDPCs, while integrins are present.

### 3.4 Ang1 promotes survival and proliferation in HFDPCs via the integrin α5β1 receptor

**Figure 4:**
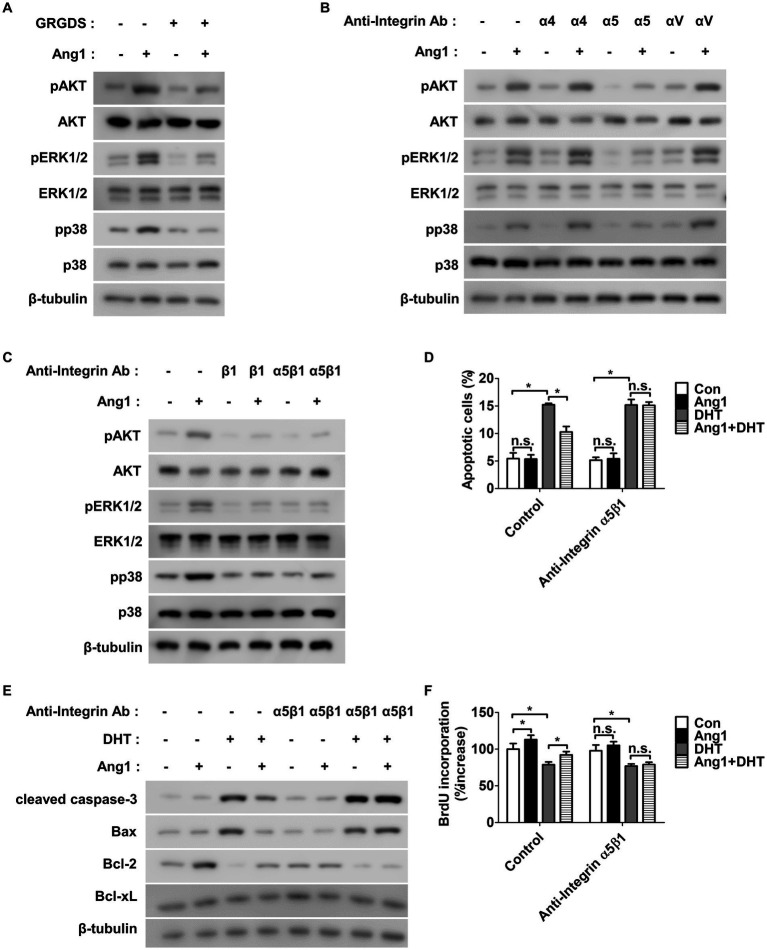
Ang1 promotes survival and proliferation of HFDPCs via integrin α5β1 receptor. **(A)** HFDPCs preincubated with GRGDS (0.5 mg/mL) for 1 h and then treated with Ang1 (300 ng/mL) for 30 min. Western blot analysis for pAKT, AKT, pERK1/2, ERK1/2, pp38, and p38 were performed on lysates obtained from HFDPCs. β-tubulin was used as a loading control. **(B,C)** HFDPCs preincubated with various integrin-blocking antibodies (5 μg/mL, α4, α5, αv, β1, α5β1) for 1 h and then treated with Ang1 (300 ng/mL) for 30 min. Western blot analysis for pAKT, AKT, pERK1/2, ERK1/2, pp38, and p38 were performed on lysates obtained from HFDPCs. β-tubulin was used as a loading control. **(D–F)** HFDPCs preincubated with integrin α5β1-blocking antibody (5 μg/mL) for 1 h and then treated with Ang1 (300 ng/mL) and/or DHT (2 μM) for 48 h. **(D)** Apoptosis of HFDPCs was analyzed using annexin-V/PI staining and flow cytometry. Bar graph represents mean ± SD (*n* = 3). n.s, not significant. *p < 0.05 by two-way ANOVA**. (E)** Western blot analysis was performed on lysates from HFDPCs to detect cleaved caspase-3, Bax, Bcl-2, and Bcl-xL. β-tubulin was used as a loading control. **(F)** Cell proliferation of HFDPCs was determined by 5′-bromodeoxy-2′-uridine (BrdU) proliferation ELISA. Bar graph represents mean ± SD (*n* = 5). n.s, not significant. *p < 0.05 by two-way ANOVA.

Since Tie-2 is absent in HFDPCs and multiple integrins are expressed, it was hypothesized that Ang1 contributes to survival and proliferation via these integrins, with an aim to identify the specific integrin involved. To determine whether Ang1 promotes the survival and proliferation of HFDPCs through integrins, the Gly-Arg-Gly-Asp-Ser (GRGDS) peptide was utilized to inhibit integrins that recognize the Arg-Gly-Asp (RGD) sequence. GRGDS inhibited the activation of Akt and ERK1/2, which contribute to Ang1-induced survival and proliferation in HFDPCs, while also reducing p38 activation. (Figure 4A). Next, various *α* integrin neutralizing antibodies were used to identify the specific integrin involved in Ang1-induced survival and proliferation in HFDPCs. Among the tested neutralizing antibodies, only integrin α5 inhibited Ang1-induced phosphorylation of AKT, ERK1/2, and p38 (Figure 4B). Since integrin α5 can form a heterodimer with integrin β1, a neutralizing antibody for integrin β1 and an integrin α5β1 neutralizing antibody were used to investigate whether Ang1 promotes survival and proliferation through integrin α5β1 in HFDPCs. The neutralizing antibodies for integrin β1 and integrin α5β1 inhibited Ang1-induced phosphorylation of AKT, ERK1/2, and p38 in HFDPCs (Figure 4C). Furthermore, the integrin α5β1 neutralizing antibody completely blocked Ang1-mediated survival under DHT treatment in HFDPCs (Figure 4D). Additionally, it fully prevented the Ang1-induced decrease in cleaved caspase-3 and pro-apoptotic Bax levels, while also inhibiting the increase in anti-apoptotic Bcl-2 levels under DHT treatment in HFPDCs (Figure 4E and Supplementary Figure 3A). The integrin α5β1 neutralizing antibody also fully inhibited Ang1-mediated proliferation under DHT treatment in HFPDCs (Figure 4F). These results indicate that Ang1 mediates survival and proliferation in HFDPCs through integrin α5β1.

## 4 Discussion

Androgenetic alopecia is the most common form of hair loss worldwide, characterized by the progressive miniaturization of terminal hairs after puberty. It affects over 80% of men and nearly 50% of women by the age of 70, with prevalence varying among ethnic groups—White individuals being the most affected, followed by Asians and Black individuals (41). Androgenetic alopecia is primarily driven by DHT, a potent androgen derived from testosterone through the action of 5α-reductase. Upon binding to androgen receptors in hair follicles, DHT promotes miniaturization by damaging DPCs, which are essential for hair follicle maintenance and cycling (42–44). This leads to the transformation of thick, pigmented terminal hairs into fine, vellus hairs and eventually results in follicular atrophy and visible hair loss.

Previous studies have demonstrated that DHT induces apoptosis in DPCs by upregulating pro-apoptotic factors, including cleaved caspase-3 and Bax, while downregulating anti-apoptotic proteins such as Bcl-2 (45). In line with these findings, our study showed that DHT increased apoptosis and suppressed proliferation in HFDPCs. Importantly, Ang1 significantly reversed these effects by restoring cell viability, inhibiting apoptosis, and promoting proliferation (Figures 1B–E), highlighting its potential as a protective agent against androgen-induced cellular damage.

Ang1, a well-characterized member of the angiopoietin family, plays a crucial role in angiogenesis, vascular stabilization, and endothelial cell survival. It typically signals through the Tie-2 receptor and integrins such as α5β1, αvβ3, and αvβ5 (27). In endothelial cells, Ang1-Tie-2 interaction activates downstream pathways, including PI3K/AKT and MAPK/ERK, which mediate cell survival and anti-inflammatory responses. However, our data revealed that Tie-2 is not expressed in HFDPCs (Figures 3A,B and Supplementary Figure 2A), suggesting that Ang1 acts through an alternative mechanism in these cells. Instead, we observed that HFDPCs express a variety of integrin subunits, including α4, α5, αv, β1, β3, β4, and β5 (Figures 3C,D and Supplementary Figure 2B), consistent with previous reports on DPCs (46, 47). Functional assays identified integrin α5β1 as the key mediator through which Ang1 enhances both survival and proliferation under DHT-induced stress (Figures 4D–F). These results establish integrin α5β1 as a novel conduit for Ang1 signaling in HFDPCs, independent of the Tie-2 receptor.

To further contextualize these findings, it is noteworthy that hair follicle growth is also regulated by additional survival and metabolic pathways such as autophagy. Recent studies have demonstrated that small molecules capable of activating autophagy stimulate hair regeneration (48), and isoquercitrin has been shown to promote hair growth through autophagy and angiogenesis via the AMPK–IGF-1R axis (49). While our study primarily focused on the PI3K/AKT and MAPK/ERK cascades downstream of integrin α5β1, it is plausible that Ang1-mediated signaling may interface with autophagy-related mechanisms, thereby further contributing to the protection and regeneration of dermal papilla cells.

While our findings emphasize the integrin-mediated effects of Ang1 in DPCs, it is important to consider the broader biological context of angiopoietin signaling. Although Tie-1 and Tie-2 were not expressed in HFDPCs, these receptors are critical regulators of vascular stability and remodeling, particularly in endothelial cells. Tie-1, unlike Tie-2, does not bind angiopoietins directly but modulates Tie-2 activity via heterodimerization and conformational regulation (27). Tie-1 is predominantly expressed under basal vascular conditions and may act as a negative regulator of Tie-2 activation (50). However, under pathological conditions such as hypoxia or inflammation, Tie-1 becomes phosphorylated and can either enhance or inhibit Tie-2 signaling depending on the context. Studies have shown that Tie-1 deficiency leads to increased vascular permeability and compromised endothelial integrity (28, 40). Our previous work demonstrated that hypoxia-induced phosphorylation of Tie-1 attenuates Ang1–Tie-2 signaling, underscoring its role as a dynamic, context-dependent modulator of vascular function (28).

Given that the dermal papilla is located within a highly vascularized microenvironment, Ang1 may also indirectly contribute to hair follicle health by promoting angiogenesis and vascular stabilization in adjacent endothelial cells via the Tie-1–Tie-2 axis. Vascular supply is essential for initiating and maintaining the anagen phase of the hair growth cycle (51–53), and reduced perifollicular vascularization has been implicated in androgenetic alopecia pathogenesis (54). Therefore, Ang1 may serve dual functions: (1) directly protecting DPCs through integrin α5β1-mediated signaling and (2) indirectly supporting follicular viability by enhancing the surrounding vascular network.

For comparison, minoxidil—the most widely used FDA-approved treatment for androgenetic alopecia—was originally developed as an antihypertensive agent. Its hair growth–promoting effect is attributed to increased perifollicular blood flow and VEGF induction, thereby enhancing angiogenesis (55, 56). Similarly, Ang1 is a potent angiogenic factor capable of promoting microvascular remodeling and endothelial stabilization (27, 57). Although our study did not directly assess Ang1-induced angiogenesis, it is plausible that Ang1 contributes to hair regeneration not only by protecting DPCs but also by facilitating vascular support within the follicular niche. This dual action may offer a mechanistic advantage over current therapies.

In conclusion, our study demonstrates that Ang1 counteracts the detrimental effects of DHT on HFDPCs by suppressing apoptosis and restoring proliferation via PI3K/AKT and MAPK/ERK1/2 signaling pathways. These effects are mediated through integrin α5β1, independently of the classical Tie-2 receptor. While Tie-1 and Tie-2 are not expressed in HFDPCs, their roles in regulating endothelial stability remain essential to the *in vivo* follicular microenvironment. Future in vivo studies incorporating endothelial–mesenchymal interactions will be important to fully elucidate whether Ang1’s direct cellular effects are complemented by its vascular functions, potentially offering a comprehensive strategy for the treatment of androgenetic alopecia.

## Data Availability

The raw data supporting the conclusions of this article will be made available by the authors, without undue reservation.
