# Supplementary material for: Angiopoietin-1 induces survival and proliferation of hair follicle dermal papilla cells through integrin α5β1 signaling
Source: Front Med (Lausanne). 2025 Sep 24;12:1649763. doi: 10.3389/fmed.2025.1649763 (PMC12504495; doi:10.3389/fmed.2025.1649763)
Supplement: Supplementary file 1 [file Data_Sheet_1.PDF]

Supplementary Material

Angiopoietin-1 induces survival and proliferation of hair follicle dermal papilla cells through integrin α5β1 signaling

Jang-Hyuk Yun [1,*]

[1]College of Veterinary Medicine and Institute of Veterinary Science, Kangwon National University, Chuncheon, Gangwon, 24341, Republic of Korea

*** Correspondence:**
Jang-Hyuk Yun
yunjh@kangwon.ac.kr

1.1 Supplementary Figures

A

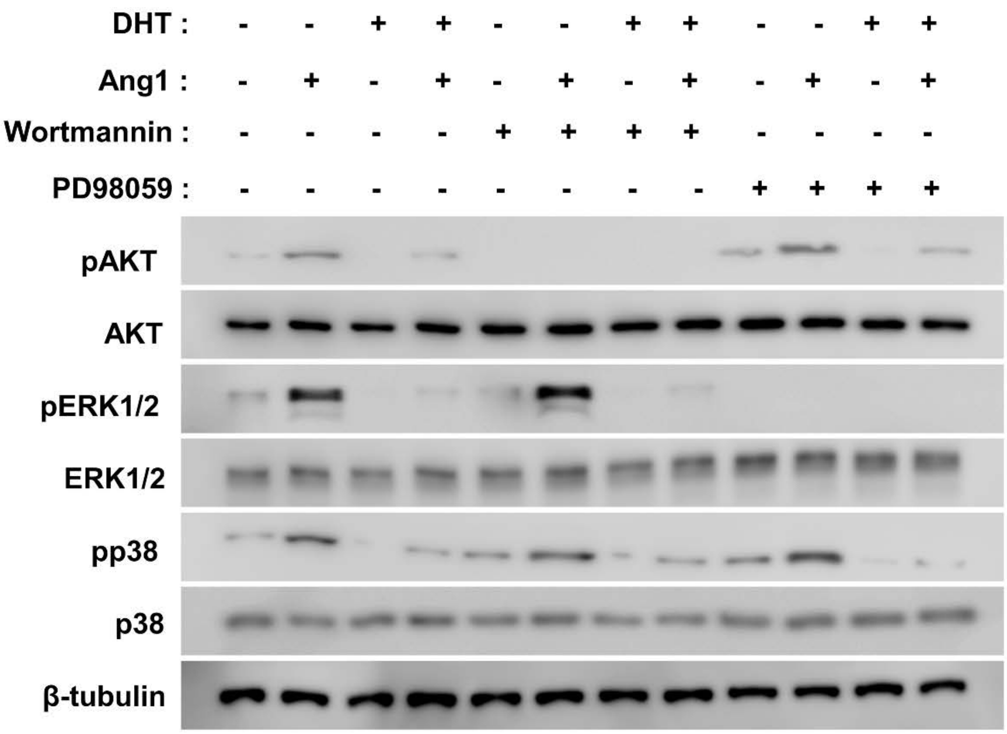

B

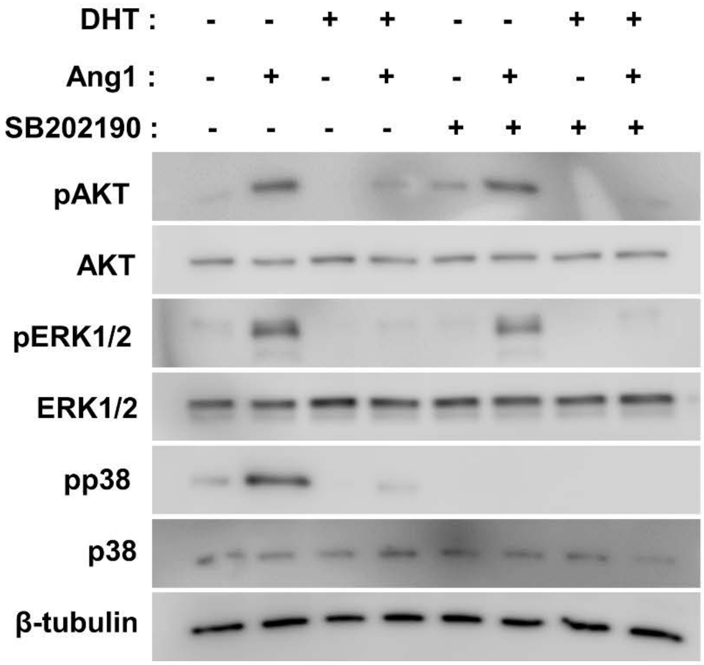

Supplementary Figure 1. Wortmannin, PD98059, and SB202190 completely block AKT, ERK1/2, and p38 phosphorylation induced by Ang1, respectively, in HFDPCs. (A-B) HFDPCs were preincubated with the Wortmannin (1 μM), PD98059 (25 μM), or SB202190 (10 μM) for 1 h and treated with Ang1 (300 ng/ml) and/or DHT (2 μM) for 30 min. Western blot analysis for phospho-AKT (pAKT), AKT, phospho-ERK1/2 (pERK1/2), ERK1/2, phospho-p38 (pp38), and p38 were performed on lysates obtained from HFDPCs. β-tubulin used as a loading control.

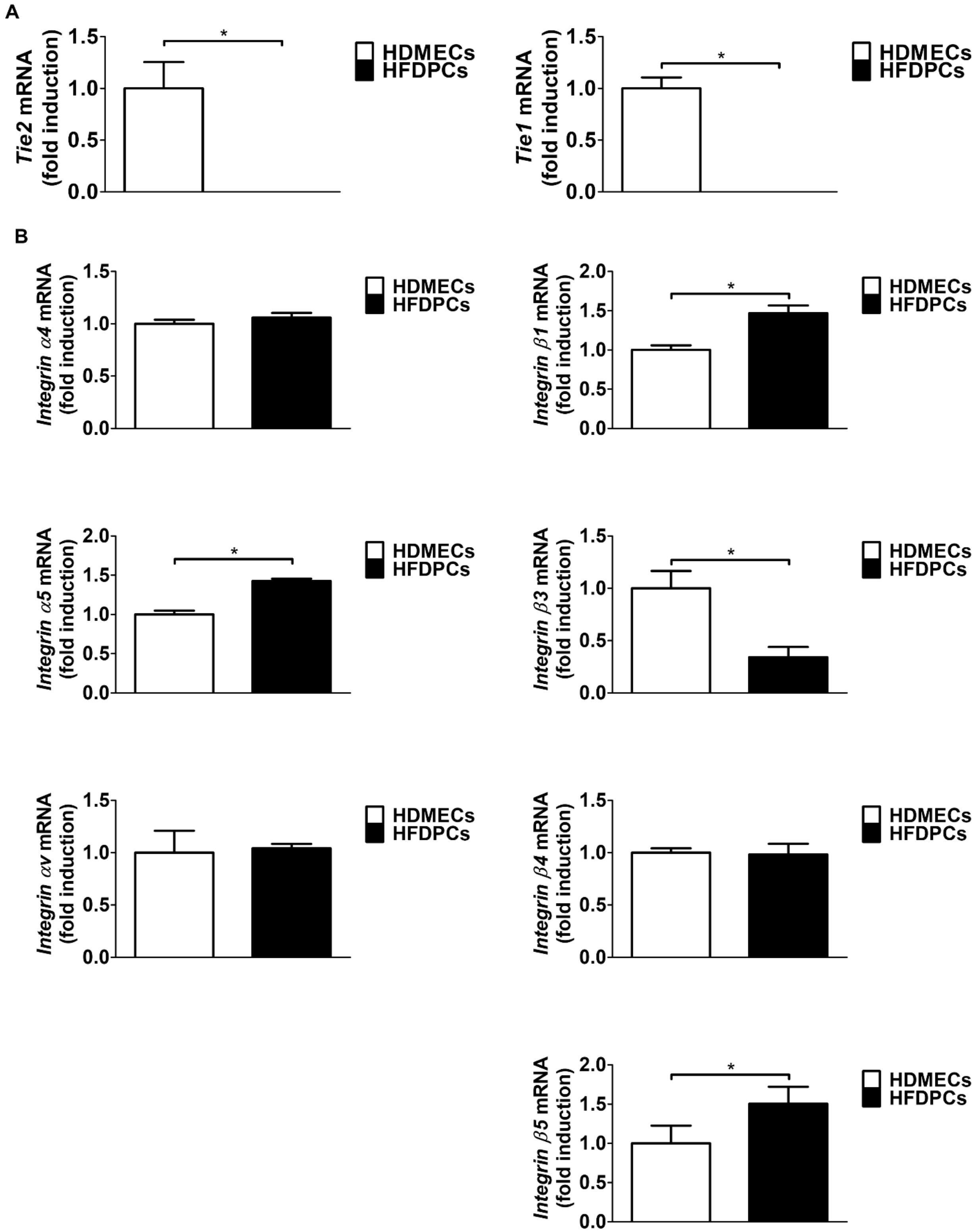

Supplementary Figure 2. HFDPCs exhibit low levels of Tie-2 receptor mRNA expression, while expressing various integrin receptor mRNAs. (A-B) qRT-PCR was performed to assess the mRNA

expression levels of Tie-2, Tie-1, and integrins α4, α5, αv, β1, β3, β4, and β5 in RNA isolated from HFDPCs and HDMECs Bar graph represents mean ± SD (*n* = 3). **P* < 0.05 by Student *t* test.

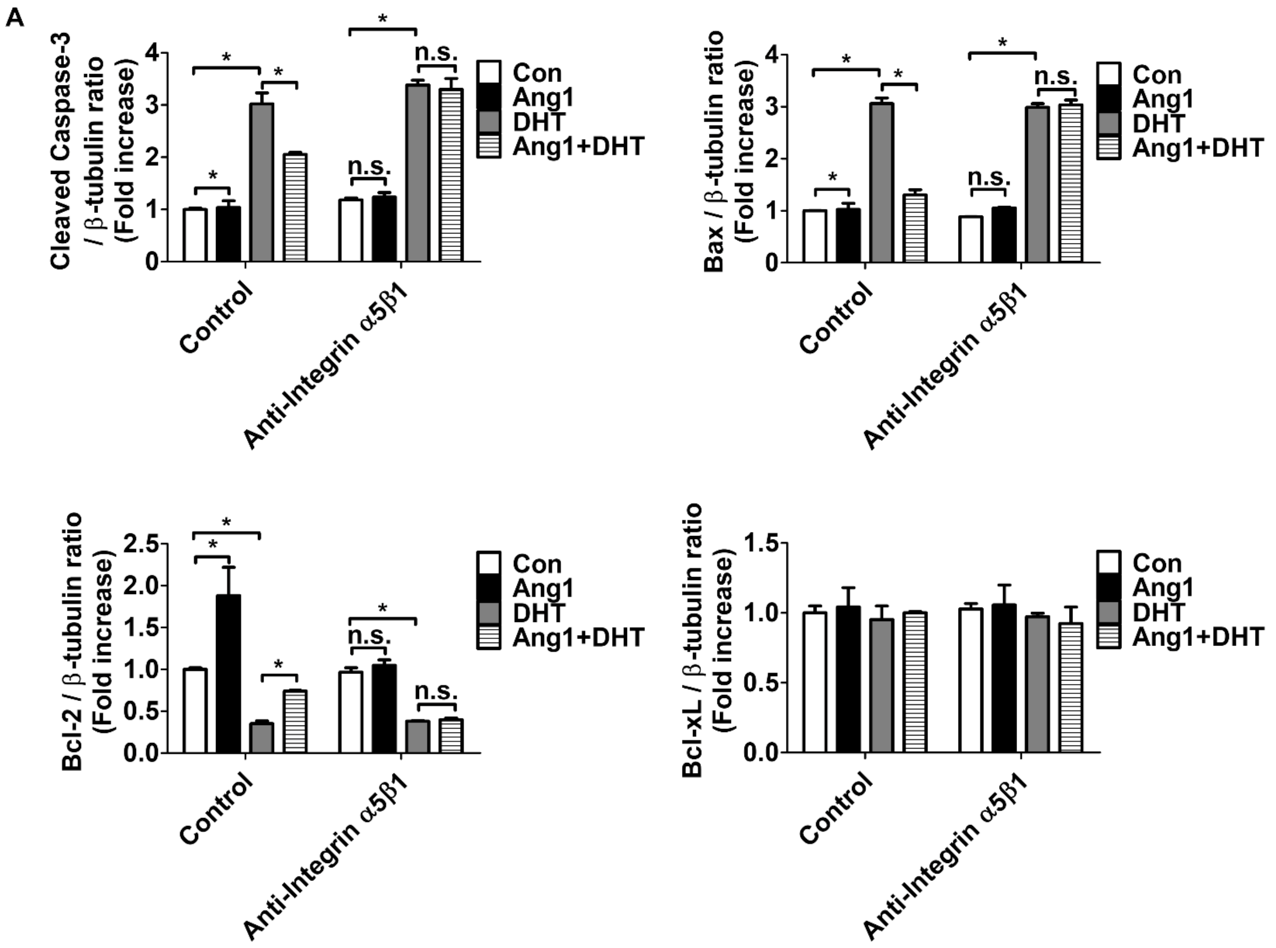

Supplementary Figure 3. Ang1 contributes to cell survival through the integrin α5β1 receptor in HFDPCs. (A) Quantitative densitometric analysis in Figure 4E to calculate the ratio of each protein to β-tubulin (n = 3). n.s, not significant. *P < 0.05 by two-way ANOVA**.**
